# Quantitative analysis of heart rate variability parameter and mental stress index

**DOI:** 10.3389/fcvm.2022.930745

**Published:** 2022-07-25

**Authors:** Jiasai Luo, Guo Zhang, Yiwei Su, Yi Lu, Yu Pang, Yuanfa Wang, Huiqian Wang, Kunfeng Cui, Yuhao Jiang, Lisha Zhong, Zhiwei Huang

**Affiliations:** ^1^Chongqing Key Laboratory of Photoelectronic Information Sensing and Transmitting Technology, Chongqing University of Posts and Telecommunications, Chongqing, China; ^2^School of Medical Information and Engineering, Southwest Medical University, Luzhou, China; ^3^State Key Laboratory of Bioelectronics, Southeast University, Nanjing, China; ^4^Central Nervous System Drug Key Laboratory of Sichuan Province, Luzhou, China

**Keywords:** heart rate variability, electrocardiogram, mental stress index, body area networks, quantitative analysis

## Abstract

**Background:**

Cardiovascular disease not only occurs in the elderly but also tends to become a common social health problem. Considering the fast pace of modern life, quantified heart rate variability (HRV) indicators combined with the convenience of wearable devices are of great significance for intelligent telemedicine. To quantify the changes in human mental state, this article proposes an improved differential threshold algorithm for R-wave detection and recognition of electrocardiogram (ECG) signals.

**Methods:**

HRV is a specific quantitative indicator of autonomic nerve regulation of the heart. The recognition rate is increased by improving the starting position of R wave and the time-window function of the traditional differential threshold method. The experimental platform is a wearable sign monitoring system constructed based on body area networks (BAN) technology. Analytic hierarchy process (AHP) is used to construct the mental stress assessment model, the weight judgment matrix is constructed according to the influence degree of HRV analysis parameters on mental stress, and the consistency check is carried out to obtain the weight value of the corresponding HRV analysis parameters.

**Results:**

Experimental results show that the recognition rate of R wave of real-time 5 min ECG data collected by this algorithm is >99%. The comprehensive index of HRV based on weight matrix can greatly reduce the deviation caused by the measurement error of each parameter. Compared with traditional characteristic wave recognition algorithms, the proposed algorithm simplifies the process, has high real-time performance, and is suitable for wearable analysis devices with low-configuration requirements.

**Conclusion:**

Our algorithm can describe the mental stress of the body quantitatively and meet the requirements of application demonstration.

## Introduction

With the influence of people's habits, diet, and environment, the death rate of cardiovascular diseases such as hypertension, coronary heart disease, and heart disease increases rapidly, and the clinical manifestations are hemiplegia, cerebral infarction, stroke, and even sudden death (1). Heart rate decreases with age. Cardiovascular disease not only occurs in the elderly but also tends to be younger (2). Heart rate variability (HRV) refers to the variation of heartbeat cycle and is a research hotspot in electrocardiogram (ECG) signal processing field in recent years (3). It contains information about the regulation of neurohumoral factors in the cardiovascular system and is a specific quantitative indicator reflecting the regulation of autonomic nerves in the heart (4). It is also the most accurate and sensitive detection indicator to judge whether patients with diabetes are accompanied by autonomic nervous system damage (5). By studying the variation of heart rate, we can reflect the influence of nervous system on cardiovascular activity. It is of great clinical immune diseases (6), rehabilitation and intensity design (7), surgical risk assessment (8), and intelligent telemedicine (9). At present, three methods, namely, resting-state HRV, task HRV, and variable HRV, are used to measure HRV. The measurement of HRV in resting state mainly refers to the way of collecting HRV in the quiet state. The measurement of HRV in the task investigates the differences in HRV of subjects under different task states. The variable HRV investigates the change in HRV, such as the change in HRV before and after a task or operation, in order to infer the effect or effect of the task or operation. Luque-Casado's research showed that the cognitive workload under execution conditions changed with the change in tasks (10). Sustained attention is a key process affecting HRV, and there is a separation between subjective and objective cognitive workload.

There are two main methods to analyze HRV, namely, time-domain analysis and spectrum analysis. In time-domain analysis, the time-domain parameters commonly used include standard deviation of normal to normal (SDNN) and root mean square successive difference (RMSSD). SDNN is generally obtained by 24 h dynamic ECG, which reflects the regulation ability of autonomic nervous system, and further reflects an individual's stress ability and resistance to pressure. RMSSD is used to assess the regulatory function and activity of the cardiac vagus nerve or parasympathetic nerve.

The spectral analysis of HRV transforms the time series of R–R interval to the frequency by mathematical transformation method, forms the spectrum curve, and analyzes the shape of the spectrum curve. Spectral analysis is usually performed at high frequency (HF, 0.15–0.40 Hz) and low frequency (LF, 0.04–0.15 Hz). HF describes parasympathetic nerve activity level, whereas LF is a sympathetic nerve activity characteristic indicator, and its ratio is negatively correlated with albumin level (11).

HRV index can be used to identify sympathetic and parasympathetic nerve activity, which can help patients with cardiovascular disease to make accurate clinical warning and take a good treatment plan. Penttila et al. (12) evaluated the applicability of these four different parameters in cardiac vagus outflow and demonstrated that these parameters are more suitable for measuring cardiac vagal outflow during free breathing. La Rovere et al. (13) quantified HRV by measuring the SDNN R–R interval with a large amount of data, which might contribute to post-infarction risk stratification. Rossi et al. (14) applied ultra-short HRV to research the influence of data loss caused by motion artifacts, which played a significant role in obtaining reliable HRV signals. After controlling for environmental and personal confounding factors, Tang et al. (15) used a linear mixed-effects model to analyze the above frequency-domain and time-domain parameters, and proved that temperature changes on the day of onset might significantly reduce cardiac autonomic nervous function. Yang et al. (16) confirmed that lower HRV was associated with a higher risk of all-cause and cardiovascular death in hemodialysis population, and that lower SDANN and LF/HF were the predictors of both all-cause and cardiovascular mortality.

HRV analysis is an important means to evaluate the state of autonomic nervous system regulating the cardiovascular system. Quantitative evaluation can directly reflect the mental state of patients, which is helpful for the evaluation of psychiatric treatment programs and detection of specific drug effects. Sripanidkulchai et al. (17) studied the effects of standard Kaempferia Parviflora (KP) extracted on physical fitness and HRV parameters of adolescent sports school students in a randomized double-blind controlled trial. The experiment proved that KP extract had an anti-stress effect on HRV parameters, which can promote its application in sports training and exercise. Yoo et al. (18) evaluated the relationship between stress measured by HRV and academic achievement of medical students during their internship.

In this article, the recognition of R characteristic wave for ECG signal, HRV parameter calculation, and mental stress (MS) evaluation was studied. The detection and recognition of the waveform is the prerequisite to judging the parameters of ECG signal. The detection and recognition of QRS waveform is the basis of ECG signal detection. If there is error detection and missing detection phenomenon of QRS waveform, it will certainly affect the judgment of P wave and T wave, and also affect the result of disease classification of ECG signal. To solve this problem, an improved difference threshold algorithm is proposed to detect R wave. The traditional difference threshold algorithm is improved in two aspects, starting point and sliding window width, to improve the accuracy of R-wave recognition. The evaluation model of MS is constructed by analytic hierarchy process (AHP), and it is verified that the algorithm can quantitatively describe the MS of the body through comparative experiments.

## Materials and methods

### Differential threshold R-wave detection algorithm

**Figure 1:**
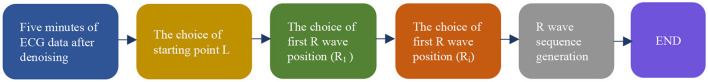
Flowchart of R-wave detection.

QRS-wave group is the most unique and easily recognized characteristic wave in ECG signal. The characteristics of R wave in wave group are particularly obvious, and the change in rising slope and falling slope is most obvious. Differential threshold R-wave detection algorithm uses the slope characteristics of the rise and fall of R wave to recognize the R wave of ECG signal. In this article, an improved differential threshold algorithm is proposed to improve the accuracy of R-wave recognition. The whole detection process of the proposed algorithm is shown in Figure 1.

**Figure 2:**
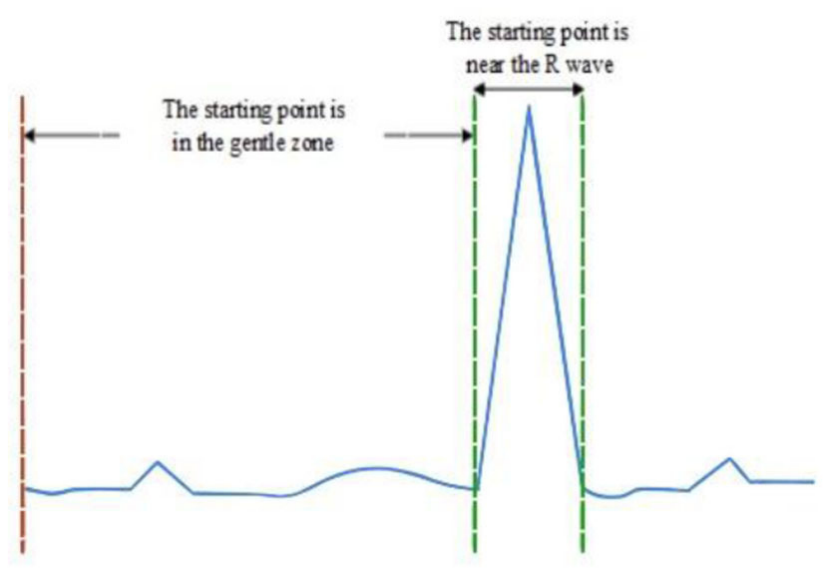
Schematic diagram of starting point selection area for R-wave detection.

The selection of initial position is very important to accurately locate all R waves. As shown in Figure 2, the starting point of R wave may occur in the following two situations. In the first case, the starting point is in the stationary part of ECG signal, which can quickly and accurately locate R wave. The second case is that the starting point is around the R wave, and when the maximum and minimum values are compared after the first-order difference, the maximum value will appear before the minimum value, which may increase the miss and fallout ratio of the R-wave recognition (19).

**Figure 3:**
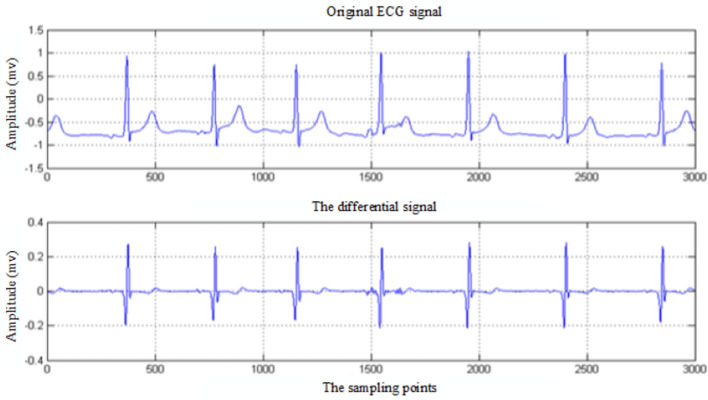
Comparison between the original ECG signal and differential signal.

To prevent the second situation mentioned above, the initial detection time of R wave should be avoided near the R wave. First, the ECG data during the period [1:t] are intercepted to find out the maximum ECG data A_max_. Then, another period ECG data during [t+1:2×t] is intercepted to find out the maximum ECG data B_max_. Finally, the value of Amax is required to compare with the value of 1.8 × B_max_. If A_max_ is greater, it indicates that the initial position is near the R wave. 2 × t will be used as the initial position for R-wave recognition. Otherwise, the initial position of R-wave detection is set as 1. After the ECG signal detection point L is selected, a segment of ECG signal X_wf_ is intercepted through the time-window function W_f_, and the signal sequence is searched to locate the position point R_1_ corresponding to the maximum value. At the same time, the first-order difference of X_wf_ will be carried out, and the comparison between the differential signal and the original ECG signal is shown in Figure 3.

**Figure 4:**
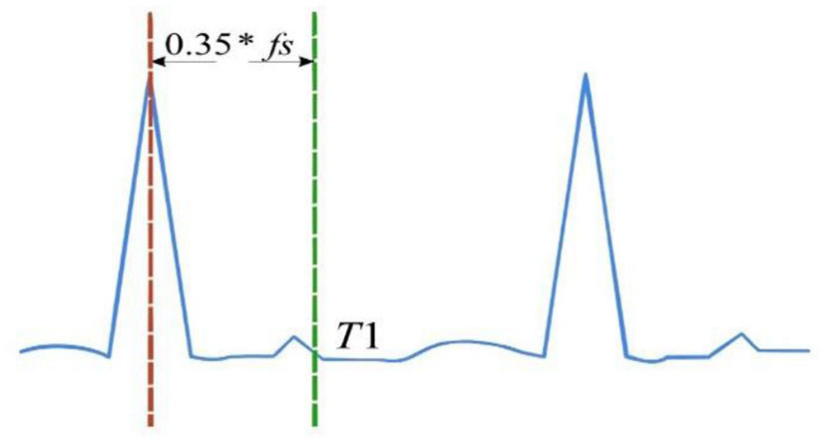
Schematic diagram of selecting the starting point for the latter R wave.

After the differential signal dif(t) of signal segment X_wf_ is calculated, the minimum and maximum values of the differential signal dif(t) are first located to correspond to the time points t_min_ and t_max_ of ECG signal. Then, the position point R′ corresponding to the maximum value of ECG signal in the time period is found. Finally, a comparison is performed to judge whether R_1_ and R′ are the same point. If they are the same point, then that point is the R-wave position. Otherwise, the smaller one is the R-wave position. Once the position of the first R wave is determined, as shown in Figure 4, the initial point L is some distance away from that position, which means that L = R + T_1_. The same process is then performed to determine the location of the second R wave. The calculated time interval between these two R waves is defined as the RR interval (20).

Similarly, after the second R-wave position is determined, the delayed T_1_ position is set as the initial point. Then, the ECG signal sequence is intercepted according to the dynamic time-window function W_f_, and the position of R wave in this segment of ECG signal is located. In this process, if R_1_ and R′ are not at the same position, the absolute values of R_1_R and R′R are, respectively, subtracted from the previous RR interval. The position point with small difference is the position point of R wave.

### Mental stress model construction based on HRV signal

**Figure 5:**
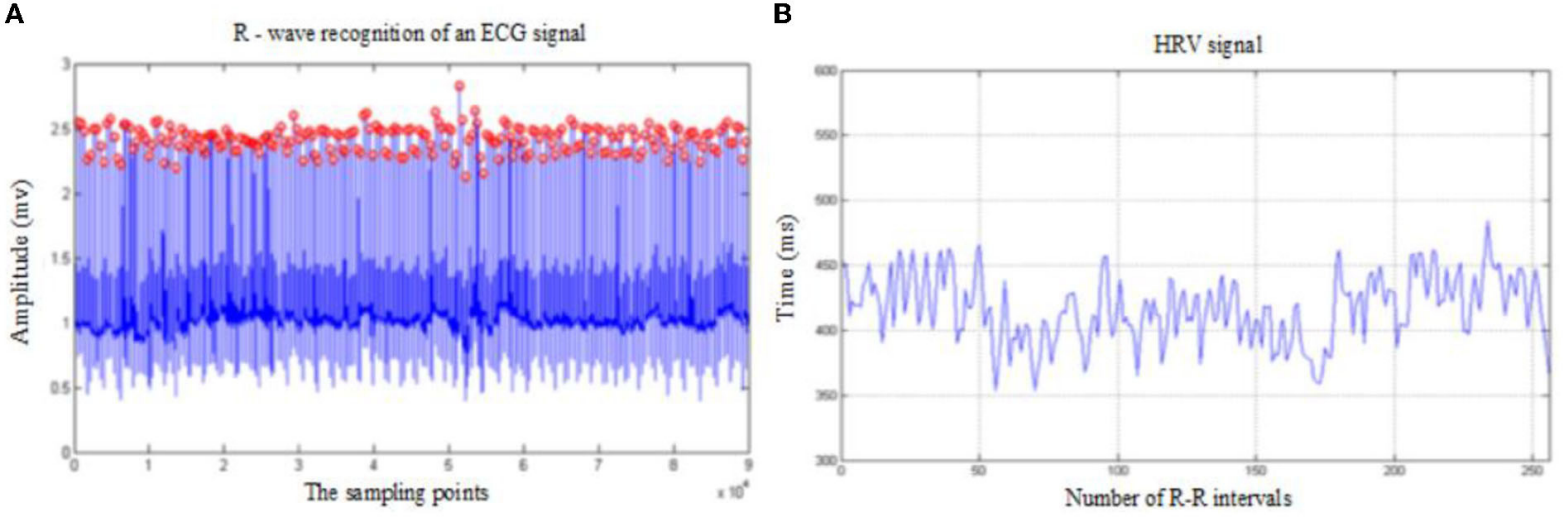
**(A)** The R-wave recognition effect diagram; **(B)** the generated HRV signal diagram.

The HRV analysis is essentially a quantitative analysis of sinus heart rate. When premature beats or severe arrhythmias appear in the signals, HRV analysis will lose its significance. Therefore, the RR interval sequence will be processed to some extent in the actual analysis process as shown in Figure 5. First, the absolute value of the difference between two adjacent RR intervals is set as *t*_*RR*_, and the critical value is set as 0.12 s. If there are 256 consecutive intervals, which are all less than the critical value when scanning the RR interval sequence, these data will be used as an HRV signal. Otherwise, it is necessary to estimate, in turn, to throw out any RR intervals in the sequence that do not satisfy the condition. Finally, the remaining RR intervals are pieced together to form HRV signals (21).

**Figure 6:**
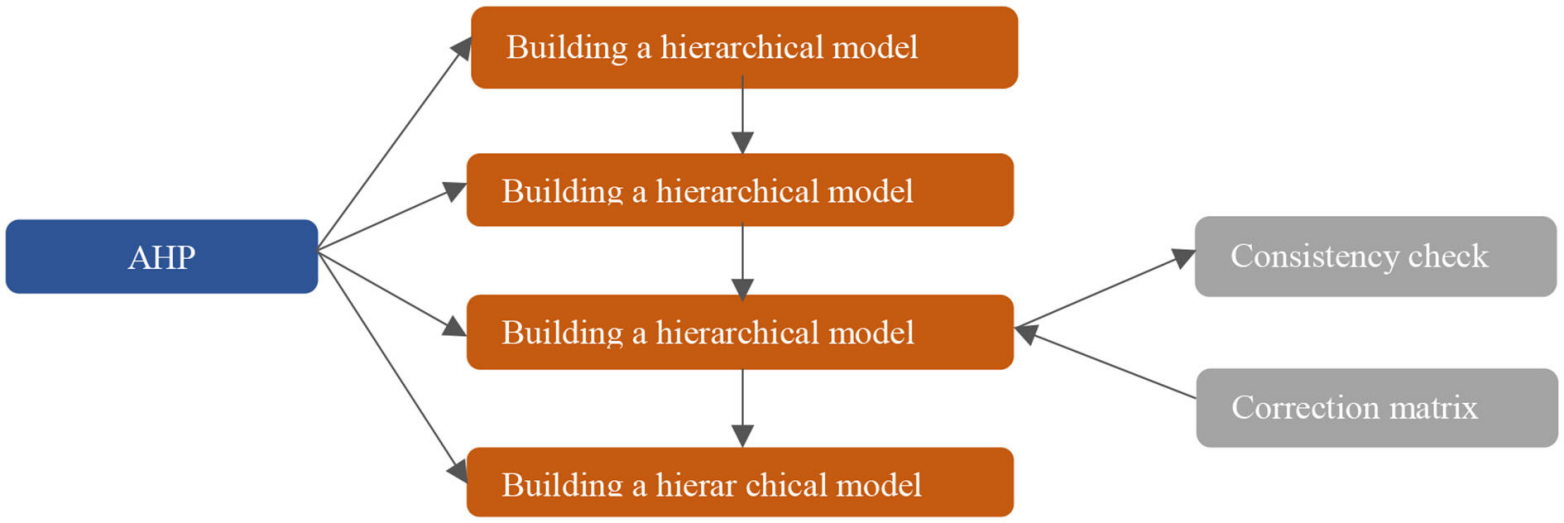
Schematic representation of the AHP.

When researchers evaluate body pressure, there is no clear judgment standard so far, and most of them determine the stress state of the subjects by observing too many indicators of HRV. In the process of HRV analysis, no matter the time-domain parameters, frequency-domain parameters, or non-linear parameters, any single parameter cannot accurately represent the MS changes of the body (22). Therefore, the AHP is proposed to conduct quantitative evaluation in this article as shown in Figure 6. By obtaining the body pressure index, testers can intuitively understand their own pressure status.

In this article, some HRV analysis parameters are selected as pressure evaluation indexes by analyzing the experimental results. Among the time-domain parameters, SDNN, PNN50, and HR are selected as pressure indicators. While TP, HF, and LF/HF are frequency-domain parameter, VAI, HRD, and HLE are non-linear parameter. The hierarchical structure of the pressure model constructed according to the pressure index is shown in Table 1. Here, G represents the target layer of the model, *G*_*i*_ represents the criterion layer of the model, and *G*_*ij*_ represents the scheme layer of the model. The body pressure value can be obtained by comprehensively weighing each level index of the hierarchical model. This process represents the MS in the form of mathematics, instead of the subjective judgment before.

**Table 1:** Pressure indicator architecture.

**Target layer (** ***G*** **)**	**Criterion layer (** ***G**_*i*_* **)**		**Scheme layer (** ***G**_*ij*_* **)**	
Body pressure index, *G*	Frequency domain	*G*_1_	LF/HF	*G*_11_
			TP	*G*_12_
	Time domain	*G*_2_	SDNN	*G*_21_
			PNN50	*G*_22_
			HR	*G*_23_
	Non-linear	*G*_3_	HLE	*G*_31_
			HRD	*G*_32_
			VAI	*G*_33_

The judgment matrix is constructed using the 1–9 scale method, which represents the ratio of the index and the importance degree of the index to the upper index in the form of numbers. The larger the ratio a/b is, the more important index a is to the upper index relative to index b. In the judgment matrix constructed using the 1–9 scale method, element *a*_*ij*_ represents the ratio of the importance of the ith element and the jth element to the upper index (23).

The 1–9 scale method is used to construct the weight judgment matrix according to the influence degree of each parameter on MS (24). The weight judgment matrix of the constructed criterion layer is as follows:

(1) $$\begin{array}{r} A\;=\;\left[\begin{array}{ccc} 1 & 4 & 5\\ 1/4 & 1 & 3\\ 1/5 & 1/3 & 1 \end{array}\right] \end{array}$$

The weight judgment matrices B_1_, B_2_, and B_3_ of frequency-domain parameters, time-domain parameters, and non-linear parameters of scheme layer G_ij_ are respectively expressed as follows:

(2) $$\begin{array}{r} B_{1}\;=\;\left[\begin{array}{cc} 1 & 3\\ 1/3 & 1 \end{array}\right]B_{2}\left[\begin{array}{ccc} 1 & 1 & 3\\ 1 & 1 & 3\\ 1/3 & 1/3 & 1 \end{array}\right]B_{3}\left[\begin{array}{ccc} 1 & 3 & 4\\ 1/3 & 1 & 2\\ 1/4 & 1/2 & 1 \end{array}\right] \end{array}$$

According to the above analysis, the weight value of each element in set *G* = {*G*_1_, *G*_2_, *G*_3_} is expressed as β = {β_1_, β_2_, β_3_}. The weight value of each element in the parameter set *G*_1_ = {*G*_11_, *G*_12_} in the frequency domain is expressed as λ_1_ = {λ_11_, λ_12_}. The weight value of each element in the parameter set *G*_2_ = {*G*_21_, *G*_22_, *G*_23_} in the time domain is expressed as λ_2_ = {λ_21_, λ_22_, λ_23_}. Similarly, the weight value of each element in the non-linear parameter set *G*_3_ = {*G*_31_, *G*_32_, *G*_33_} is expressed as λ_3_ = {λ_31_, λ_32_, λ_33_}. The body pressure evaluation model of the criterion layer, expressed as *Z*_*G*_*i*__ = *f*(*G*_*ij*_, λ_*ij*_), is constructed by combining the weight of each parameter. Here, *G*_*ij*_ is HRV analysis parameter value, and λ_*ij*_ is the corresponding weight value. The calculation method of criterion layer is as follows:

(3) $$\begin{array}{r} Z_{G1}=\left(\lambda_{11}\times \frac{G_{11}}{15}+\lambda_{12}\times \frac{G_{12}}{9000}\right)\times 100\% \end{array}$$

(4) $$\begin{array}{r} Z_{G_{2}}=(\lambda_{21}\times \frac{200-G_{21}}{200}+\lambda_{22}\times \frac{60-G_{22}}{60}+\lambda_{23}\\ \;\times \frac{G_{23}}{100})\times 100\% \end{array}$$

(5) $$\begin{array}{r} Z_{G3}=(\lambda_{31}\times \frac{G_{31}}{10}+\lambda_{32}\times \frac{0.4-G_{32}}{0.4}+\lambda_{33}\\ \;\times \frac{10-G_{33}}{10})\times 100\% \end{array}$$

After the value of the secondary model *Z*_*G*_*i*__ is calculated, the body pressure value is calculated by combining the weight of the criterion layer β_*i*_. The calculation formula of pressure index of the target layer model is shown in the following formula:

(6) $$\begin{array}{r} Z=\overset{k}{\underset{i=1}{\sum }}100\beta_{i}\times Z_{G_{i}} \end{array}$$

## Results

### Experimental test platform

**Figure 7:**
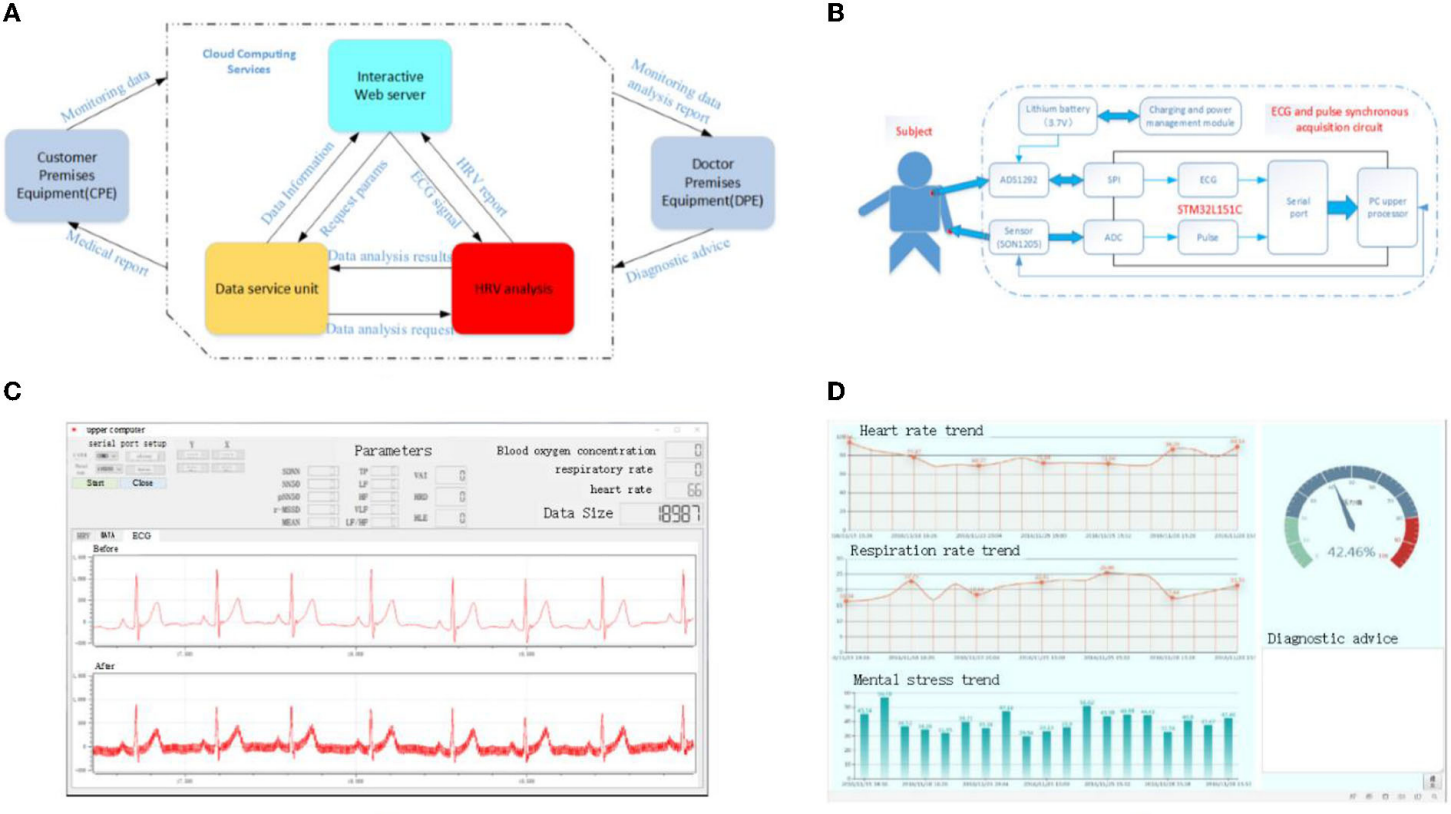
Schematic representation of the experimental test platform for HRV. **(A)** Overall structure diagram of the server, **(B)** schematic diagram of the customer premises equipment, **(C)** data acquisition interface of upper computer, and **(D)** electronic medical record interface.

The test platform in this article is a wearable sign monitoring system constructed based on BAN technology. The overall design structure block diagram of the system is shown in Figure 7A. It includes a three-layer structure of customer-premises equipment (CPE), cloud computing services, and doctor-premises equipment (DPE). The ECG signal is collected by the client and sent to a remote data center to obtain a diagnostic report. On the one hand, the remote data center receives the collected user data, calculates the HRV signal, and transmits the calculation results to the doctor. On the other hand, it receives the medical report transmitted by the doctor and feeds it back to the client. The doctor side will present the final HRV data calculation results, and the doctor can control the patient's status in real time for timely diagnosis and treatment advice. The hardware circuit diagram of the CPE in this experiment is shown in Figure 7B. The device simultaneously collects ECG and pulse signals of the human body. The ECG signal is collected by CM5 bipolar chest lead, with two lead wires as positive pole LA and negative pole RA. The electrode is attached to the human body, and the ECG signal is transmitted to the acquisition circuit board through the lead wire. The ADS1292R chip amplifies the analog ECG signal, converts A/D into digital signal, and sends it to the MCU processing circuit through SPI interface. The microcontroller assembles the collected ECG and pulse signals into frames, sends them to the USB-serial circuit, and then the computer. At this point, the synchronous collection of ECG and pulse is completed. The data collection interface of the upper computer is shown in Figure 7C. The HRV time-domain, frequency–domain, and non-linear data can be presented by calculation. Figure 7D is the doctor-side diagnosis and treatment interface, which can intuitively display the patient's current MS status and query the historical changes of MS, and provide the best diagnosis and treatment suggestions accordingly.

The test data are measured in cooperation with Southwest Medical University. The ages of subjects are between 20 and 40 years, and the data are collected for 5 min at a time. The Institution Research Ethics Board of Southwest Medical University approved this experiment, and all experiments are performed in accordance with relevant guidelines and regulations. In addition, all volunteers who provide data have agreed to use the data for publication and informed consent is obtained from all subjects.

### Verification of R-wave detection algorithm

**Figure 8:**
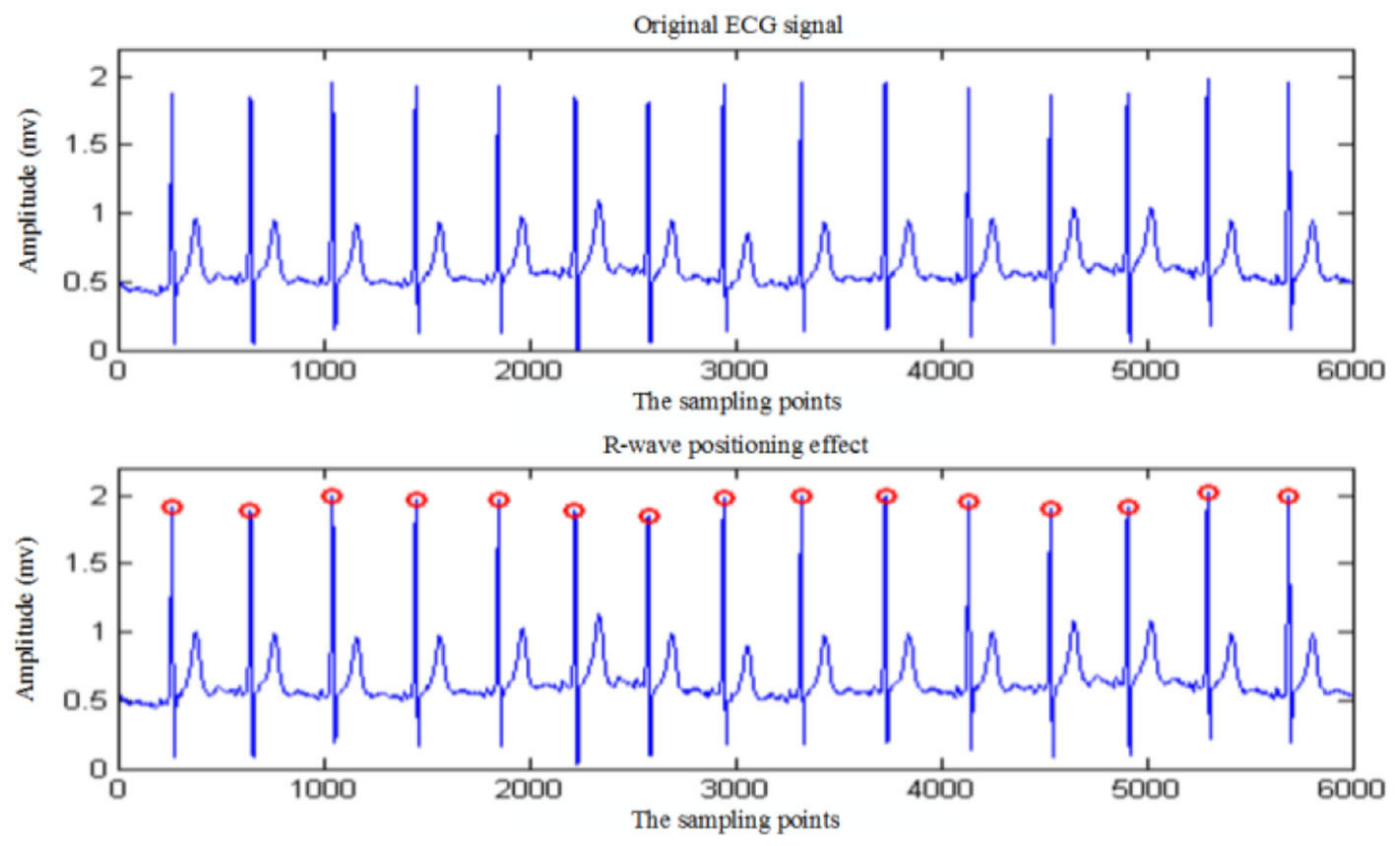
R-wave detection of ECG signal without noise interference.

**Figure 9:**
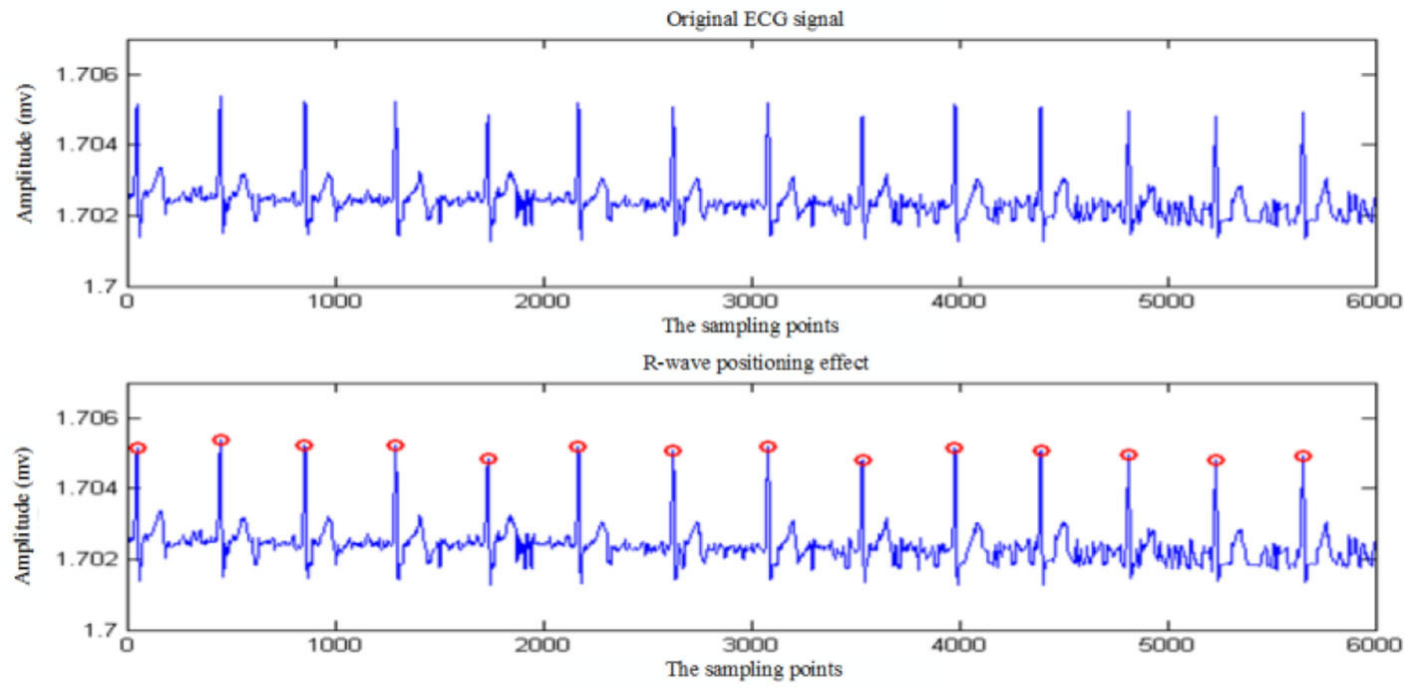
R-wave detection of ECG signal with noise interference.

To verify the accuracy and anti-jamming ability of the R-wave detection algorithm, the R-wave location simulation of ECG signals under different conditions is carried out by using this algorithm on the test platform. The positioning effect diagram of ECG signal without noise interference is shown in Figure 8, and the one with noise interference is shown in Figure 9.

Real-time collection of 5 min ECG data using the algorithm presented in this article is used to identify R wave. The simulation results show that the algorithm recognition rate of R wave reaches 100%. To further verify the accuracy of the algorithm, 30 min ECG data from the MIT/BIH ECG database is used to detect and identify R characteristic waves. Table 2 shows the statistics of the recognition rate of the R-wave recognition algorithm for partial ECG data randomly selected from the MIT/BIH database. As shown in Table 2, the average R-wave recognition rate of this algorithm for the electrical signals in the center of the MIT/BIH database is ~99.69%, which is mainly caused by excessive noise interference in the signals.

**Table 2:** R-wave detection statistics of partial ECG data in the MIT/BIH database.

**File No**.	**No. of standard QRS**	**Missing detection**	**Fault detection**	**Incorrect total**	**Recognition rate**
100	2,273	0	1	1	99.91%
102	2,187	0	2	2	99.91%
103	2,084	1	2	3	99.86%
104	2,229	1	5	6	99.73%
105	2,572	2	6	8	99.69%
107	2,137	0	5	5	99.77%
109	2,532	1	8	9	99.64%
123	1,518	2	1	3	99.80%
208	2,955	0	9	9	99.70%
210	2,650	5	8	13	99.51%
215	3363	5	7	12	99.64%
219	2,154	0	5	5	99.77%
230	2,256	2	1	3	99.87%
Total	30,910	19	60	79	99.69%

The R-wave recognition algorithm proposed in this article has the following advantages over the traditional R-wave recognition algorithm. On the one hand, the algorithm improves the R-wave recognition rate and avoids missing and wrong detection of short-range ECG signals. On the other hand, the algorithm is easy to implement and the computation is small, so it is suitable for the analysis equipment with a high real-time requirement.

### Contrastive experimental verification of mental states under different conditions

**Figure 10:**
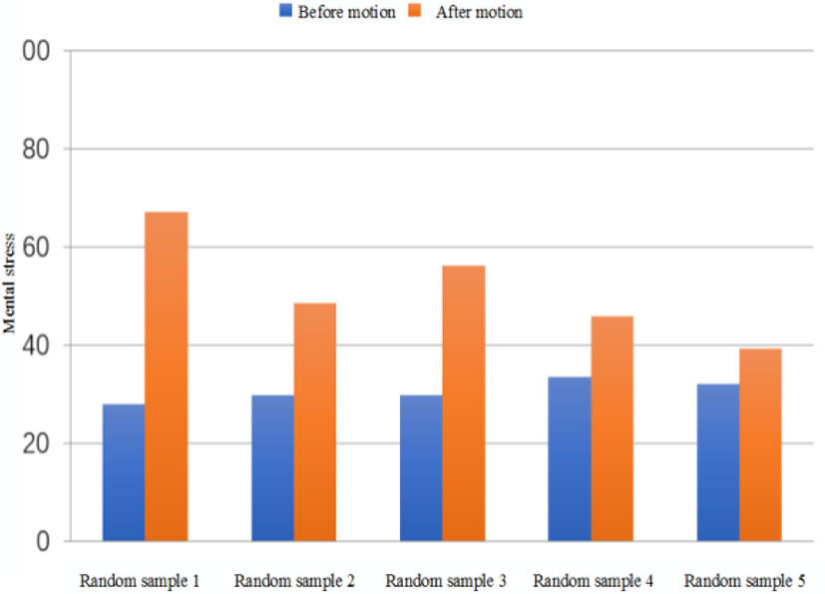
Comparison of mental stress before and after exercise for random sample.

In this article, a comparative experiment is carried out according to the difference of motion state and environmental factors. It is found that muscle tension and mental activity could increase after exercise. In this experiment, HRV analysis parameters are used to calculate and compare MS values to verify the validity of the pressure model. Table 3 shows the comparison of experimental data of five randomly selected subjects in the database in two environments. From Figure 10, we can see the calculated quantitative indexes of MS before and after exercise. It can be seen from the figure that the MS value obtained by comprehensive calculation after exercise is significantly increased compared with that before exercise, which is in line with the objective fact, indicating that this model can be used for stress assessment caused by exercise.

**Table 3:** Comparison of random experimental data and pressure index before and after exercise.

**No**.	**State**	**VAI**	**HRD**	**HLE**	**HR**	**SDNN**	**PNN50**	**TP**	**LF/HF**
1	Before	1.11	0.07	5.01	81	54.20	4.70	1,365	0.29
	After	0.73	0.07	7.48	99	35.26	1.90	1,929	10.45
2	Before	1.06	0.06	5.64	80	42.63	5.10	1,914	0.30
	After	0.88	0.05	6.71	102	21.43	1.10	3,924	3.86
3	Before	1.39	0.06	5.80	73	34.01	9.80	1,700	0.51
	After	0.89	0.06	6.35	95	24.36	1.90	2,042	7.28
4	Before	1.08	0.08	5.22	79	68.98	5.90	4,920	0.21
	After	0.03	0.05	5.26	100	63.36	1.20	6,743	2.23
5	Before	1.27	0.07	5.76	80	46.17	7.90	1,675	1.26
	After	0.91	0.07	6.21	98	30.12	2.80	2,485	2.05

Drastic changes in the environment may stress the subjects. The validity of the pressure evaluation model is verified by comparing the pressure values of the test subjects under two conditions. As we all know, virtual reality (VR) video has a certain auxiliary effect on mental illness and is being used by many psychiatrists to assist diagnosis and treatment. On account of this reason, VR video is used in this article to create different conditions for the subjects.

**Figure 11:**
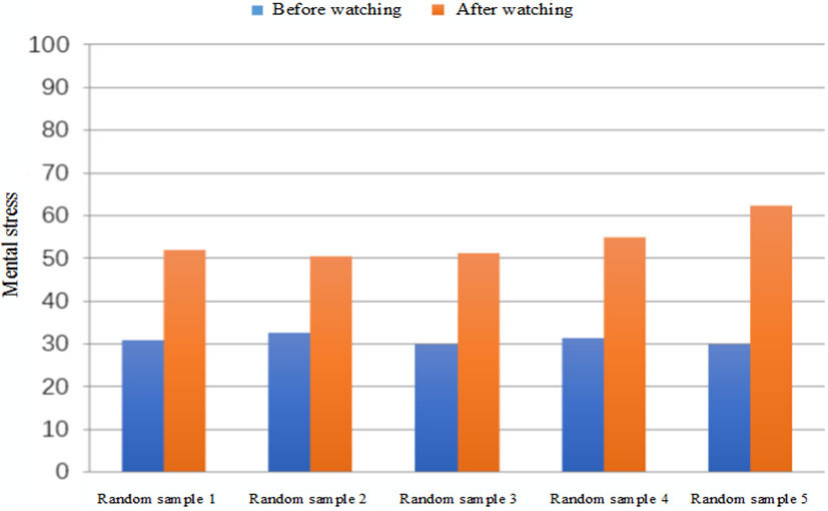
Comparison of mental stress before and after watching the video.

VR videos tested include soothing landscapes and a scary movie. First, the subject's MS is calculated and recorded while the subject is relaxed by watching the soothing landscapes. Every subject is then left alone in a dark room to watch a horror movie clip. The corresponding MS in a state of anxiety and tension is calculated and recorded. Table 4 shows the comparison of experimental data of five randomly selected subjects in the database in two conditions. Figure 11 shows the comparison of MS values.

**Table 4:** Comparison of random experimental data and pressure index before and after watching.

**No**.	**State**	**VAI**	**HRD**	**HLE**	**HR**	**SDNN**	**PNN50**	**TP**	**LF/HF**
1	Before	1.12	0.05	5.86	77	52.85	4.11	1,728	0.76
	After	0.65	0.05	7.07	90	37.44	0.98	3,415	5.25
2	Before	2.52	0.06	5.12	70	32.29	0.78	2,992	0.35
	After	1.11	0.06	7.48	97	21.58	0	4,469	3.88
3	Before	1.06	0.05	4.96	72	48.04	2.54	2,210	0.26
	After	0.84	0.03	6.71	98	34.41	0	5,264	3.94
4	Before	2.61	0.11	5.63	80	51.7	9.59	2,426	0.9
	After	1.05	0.06	6.68	87	44.42	0.78	3,262	6.5
5	Before	1.26	0.07	5.35	77	52.85	2.15	822	1.01
	After	0.65	0.05	7.27	90	25.29	0.39	3,415	8.18

According to the results of the experiment, the subjects are relaxed when watching the scenery videos, with almost no psychological or physical stress. The value of the corresponding MS is small, and the average value is ~30. After watching the scary video, the psychological activity of the subjects is greater and even produces a sense of fear. As can be seen from Figure 11, the value of MS increases significantly. Although there are individual differences, the increase is generally more than 60%, and the biggest change even reaches 106.7%. This quantitative description is capable of reflecting the changes of MS effectively brought by different stimuli to the organism.

### Error analysis of measurement data

Considering the influence of subjects, environment, and measuring instruments, there will be some errors in the actual measurement. To reduce the error, we calculate the average of multiple measurements. Table 5 shows all error rates of HRV parameters.

**Table 5:** Error rate of HRV parameters.

**No**.	**SDNN**	**pNN50**	**HR**	**LF/HF**	**TP**	**HRD**	**HLE**	**VAI**	**MS**
1	2.94%	3.82%	1.29%	23.97%	4.28%	2.95%	0.59%	0.57%	4.71%
2	5.53%	15.41%	1.41%	16.35%	1.92%	5.56%	0.42%	1.16%	7.76%
3	4.35%	4.37%	1.62%	6.09%	7.19%	5.16%	3.72%	12.68%	1.98%
4	2.81%	3.82%	0.79%	24.00%	4.27%	6.72%	1.76%	1.65%	1.24%
5	3.42%	8.41%	1.27%	24.09%	4.73%	9.06%	0.55%	5.39%	2.97%
6	11.01%	8.27%	0.67%	18.79%	6.32%	4.83%	7.26%	9.65%	4.26%
7	6.52%	17.07%	2.12%	19.00%	5.05%	2.28%	1.34%	2.82%	5.71%
8	2.99%	26.36%	2.56%	18.89%	3.37%	3.00%	2.72%	9.93%	8.74%
9	2.98%	22.50%	3.55%	18.30%	2.33%	2.98%	3.20%	1.00%	9.68%
10	4.79%	20.03%	4.42%	28.22%	36.35%	6.25%	6.53%	11.80%	9.34%
11	17.27%	13.49%	1.92%	18.86%	23.27%	13.03%	1.59%	3.84%	0.57%
12	0.16%	19.08%	1.40%	6.57%	4.96%	0.19%	3.46%	8.85%	5.77%
13	3.96%	1.55%	2.53%	3.20%	3.29%	2.98%	1.25%	7.72%	1.77%
14	14.97%	16.17%	1.14%	20.64%	10.96%	14.96%	6.60%	8.21%	3.13%
15	3.96%	0.53%	1.63%	34.48%	13.33%	3.98%	1.55%	0.39%	5.26%
16	3.24%	0.70%	0.47%	29.74%	22.32%	5.68%	2.47%	3.02%	0.86%
17	13.18%	22.17%	1.15%	25.24%	23.61%	3.41%	2.81%	3.22%	2.58%
18	5.31%	13.46%	2.44%	6.78%	10.10%	5.31%	3.07%	3.32%	0.78%
19	6.09%	15.00%	3.02%	7.45%	6.75%	5.39%	3.18%	1.90%	1.72%
20	4.27%	19.99%	1.55%	19.15%	7.92%	3.41%	2.81%	1.61%	4.10%

The result shows that the errors of SDNN in the time domain range from 0.16 to 17.27%, PNN50 range from 0.53 to 26.36%, HR range from 0.42 to 3.02%, and LF/HF range from 3.20 to 34.48%. Total power (TP) error is between 1.92 and 36.35%. The errors of relative dispersion of non-linear parameters HRD, Lyapunov index HLE, and vector angle index (VAI) are 0.19–14.96%, 0.42–7.26%, and 0.57–12.68%, respectively. The error of the final calculated MS index is between 0.57 and 9.68%, which meets the requirements of application demonstration.

## Discussion

In this study, we construct the quantitative description model of mental stress, propose an improved differential threshold method to detect, and recognize R waves of ECG signals accurately and reliably. Both these research findings shed light on the accurate HRV measurement and quantitative detection of mental stress, likely playing a role in the treatment of mental illness.

By analyzing HRV time-domain and frequency-domain indexes, researchers aim to quantitatively study the relationship between HRV indexes and sympathetic nerve activity level and special diseases. Penttila et al. (12) studied the applicability of four different measures of HRV in the assessment of cardiac vagal outflow. However, its research tended to be in the category of free breathing and could not be directly quantified. Recent research by Rovere (13) introduced Baroreflex Sensitivity (BRS) for risk stratification after infarction on the basis of traditional time-domain parameter SDNN. Based on 24 h Holter recording and rate–pressure response to intravenous phenylephrine, they unveiled that low values of either HRV (SDNN < 70 ms) or BRS (<3 ms per mmHg) carried a significant multivariate risk of cardiac mortality. However, the results of this study have great limitations, especially for patients with low BRS. Furthermore, as a time-domain parameter, SDNN has a great interference and error in the measurement process. Yang et al. (16) confirmed that the reduction in HRV was associated with a high risk of all-cause and cardiovascular death in hemodialysis population. Decreased SDANN and LF/HF were identified as predictors of both all-cause and cardiovascular mortality, while the utility of other HRV metrics requires further investigation. Tang's (15) study showed that temperature variability decreased both frequency-domain and time-domain HRV parameters, which is a possible predictor for adverse cardiac events. Consistent with these results, we found that HRV index can effectively predict the occurrence of some diseases, but also can be used as the evaluation basis of some diseases. Through the above experiments, we verified the changes of HRV indicators under different emotions and different sports states, and gave quantitative descriptions to intuitively evaluate the changes in their diseases.

To truly estimate disease status through HRV index, accurate measurement and calculation of acquisition parameters are very important. Rossi et al. (14) simulated missing values induced by motion artifacts (from 0 to 70%) in an ultra-short-time window by the random walk Gilbert burst model in 22 young healthy subjects to obtain reliable HRV parameters from device. Tang's (15) study integrated eight measurement parameters in time domain and frequency domain to ensure the reliability of calculation results. Although there is extensive evidence that multiparameter comprehensive measurement can effectively reduce measurement error and improve data reliability, we show for the first time that the comprehensive index of HRV based on weight matrix in Equation (6) can greatly reduce the deviation caused by the measurement error of each parameter as shown in Table 5.

This study has some limitations. One limitation is that the all participants are adults. Future studies could be performed in order to detect individual characteristics such as gender, age, and individual habits. Another limitation is the lack of a broader stimulus. Future studies could be performed in order to consider stimulating factor such as sadness, shock, and weightlessness.

## Conclusion

The quantitative analysis of HRV can reflect the regulation of the autonomic nervous system to the cardiovascular system. Not only can it help patients and doctors diagnose or predict cardiovascular disease, but it can also be used to assess psychological conditions such as stress. In this article, an improved differential threshold method is used to detect and recognize R waves of ECG signals. To improve the recognition rate, the traditional difference threshold method is improved through a novel algorithm for locating initial position of R wave and improved time-window function. The experimental results show that the recognition rate of R wave in 5 min ECG data collected in real time is >99%. Compared with traditional characteristic wave recognition algorithms, the proposed algorithm simplifies process, has high real-time performance, and is suitable for wearable analysis devices with low-configuration requirements. The accurate recognition of ECG signal R characteristic wave goes far toward obtaining an accurate HRV signal. The AHP is used to construct the mental stress evaluation model in this article. According to the influence degree of HRV analysis parameters on mental stress, the weight judgment matrix is constructed and the consistency is verified. Then, the weight values of the corresponding HRV analysis parameters are calculated. In the end, according to the value range of the parameters selected by the model and the ratio relationship between the parameters and mental stress, the mental stress evaluation model is constructed. The validity of the mental stress model is verified by comparing the mental stress values of the subjects in different states and different environments. The experimental results show that the mental stress model can describe the mental stress of the body quantitatively.

## Data availability statement

The original contributions presented in the study are included in the article/supplementary materials, further inquiries can be directed to the corresponding author/s.

## Ethics statement

Ethical review and approval was not required for the study on human participants in accordance with the local legislation and institutional requirements. The patients/participants provided their written informed consent to participate in this study. Written informed consent was obtained from the individual(s) for the publication of any potentially identifiable images or data included in this article.

## Author contributions

JL and GZ: writing the manuscript. YS and YL: collecting the data. YP and YW: designing the research. HW, KC, and YJ: statistical analysis. LZ and ZH: obtaining funding. All authors have read and approved the final manuscript.

## Funding

This project was funded by Central Nervous System Drug Key Laboratory of Sichuan Province, Medical Engineering and Medical Informatics Integration and Transformational Medicine of Luzhou Key Laboratory and also supported by the National Natural Science Foundation of China (No. 62171073, No. U21A20447, and No. 61971079), China Postdoctoral Science Foundation (No. 2021M693931 and No. 2021MD703941), Science and Technology Research Program of Chongqing Municipal Education Commission (KJQN202000604 and KJQN202100602), Nature Science Foundation of Chongqing (cstc2020jcyj-cxttX0002, cstc2021jscx-gksbx0051, and cstc2021jcyj-bsh0221), Science and Technology Department of Sichuan Province (2020YFQ0025, 2020YJ0151, and 2022NSFSC0508), Key Research Project of Southwest Medical University (2021ZKZD019), Project of Central Nervous System Drug Key Laboratory of Sichuan Province (200018-01SZ, 200020-01SZ, 200027-01SZ, 200028-01SZ, and 210022-01SZ), and Open Research Fund of State Key Laboratory of Bioelectronics, Southeast University (No. SKLB2022-P06).

## Conflict of interest

The authors declare that the research was conducted in the absence of any commercial or financial relationships that could be construed as a potential conflict of interest.

## Publisher's note

All claims expressed in this article are solely those of the authors and do not necessarily represent those of their affiliated organizations, or those of the publisher, the editors and the reviewers. Any product that may be evaluated in this article, or claim that may be made by its manufacturer, is not guaranteed or endorsed by the publisher.
